# A comparative study of functional MRI in predicting response of regional nodes to induction chemotherapy in patients with nasopharyngeal carcinoma

**DOI:** 10.3389/fonc.2022.960490

**Published:** 2022-08-31

**Authors:** Dawei Zhao, Xuemei Fang, Wenjun Fan, Lingling Meng, Yanrong Luo, Nanxiang Chen, Jinfeng Li, Xiao Zang, Meng Li, Xingdong Guo, Biyang Cao, Chenchen Wu, Xin Tan, Boning Cai, Lin Ma

**Affiliations:** ^1^ Department of Radiation Oncology, First Medical Center of Chinese PLA General Hospital, Beijing, China; ^2^ Department of Radiology, Characteristic Medical Center of Chinese People’s Armed Police Force, Tianjin, China; ^3^ Department of Ultrasound, Tianjin Medical University General Hospital Airport Hospital, Tianjin, China; ^4^ Affiliated Foshan Maternity & Child Healthcare Hospital, Southern Medical University, Foshan, China; ^5^ Department of Oncology, Armed Police Forces Corps Hospital of Henan Province, Zhengzhou, China; ^6^ Department of Otolaryngology, First Medical Center of Chinese PLA General Hospital, Beijing, China; ^7^ Department of Radiology, First Medical Center of Chinese PLA General Hospital, Beijing, China; ^8^ Department of Radiation Oncology, Fifth Medical Center of Chinese PLA General Hospital, Beijing, China

**Keywords:** functional magnetic resonance imaging, magnetic resonance imaging, induction chemotherapy, lymph nodes, nasopharyngeal carcinoma

## Abstract

**Purpose:**

To identify and compare the value of functional MRI (fMRI) in predicting the early response of metastatic cervical lymph nodes (LNs) to induction chemotherapy (IC) in nasopharyngeal carcinoma (NPC) patients.

**Methods:**

This prospective study collected 94 metastatic LNs from 40 consecutive NPC patients treated with IC from January 2021 to May 2021. Conventional diffusion-weighted imaging, diffusion kurtosis imaging, intravoxel incoherent motion, and dynamic contrast-enhanced magnetic resonance imaging were performed before and after IC. The parameter maps apparent diffusion coefficient (ADC), mean diffusion coefficient (MD), mean kurtosis (MK), D_slow_, D_fast_, perfusion fraction (PF), K^trans^, V_e_, and K_ep_) of the metastatic nodes were calculated by the Functool postprocessing software. All LNs were classified as the responding group (RG) and non-responding group (NRG) according to Response Evaluation Criteria in Solid Tumors 1.1. The fMRI parameters were compared before and after IC and between the RG and the NRG. The significant parameters are fitted by logistic regression analysis to produce new predictive factor (PRE)–predicted probabilities. Logistic regression analysis and receiver operating characteristic (ROC) curves were performed to further identify and compare the efficacy of the parameters.

**Results:**

After IC, the mean values of ADC, MD, and D_slow_ significantly increased, while MK, D_fast_, and K^trans^ values decreased dramatically, while no significant difference was detected in V_e_ and K_ep_. Compared with NRG, PF-pre and K^trans^-pre values in the RG were higher statistically. The areas under the ROC for the pretreatment PF, K^trans^, and PRE were 0.736, 0.722, and 0.810, respectively, with the optimal cutoff value of 222 × 10^-4^, 934 × 10^-3^/min, and 0.6624, respectively.

**Conclusions:**

The pretreatment fMRI parameters PF and K^trans^ showed promising potential in predicting the response of the metastatic LNs to IC in NPC patients.

**Clinical Trial Registration:**

This study was approved by the ethics board of the Chinese PLA General Hospital, and registered on 30 January 2021, in the Chinese Clinical Trial Registry; http://www.chictr.org.cn/showproj.aspx?proj=121198, identifier (ChiCTR2100042863).

## Introduction

Nasopharyngeal carcinoma (NPC) is an epithelial carcinoma arising from the nasopharyngeal mucosal lining, which has remarkable epidemiological features, and more than 70% of new cases are in East and Southeast Asia (1). Almost 79.1% of NPC patients present with metastatic cervical lymph nodes (LNs) once diagnosed, which is strongly correlated with distant metastasis (2). The number (3) and morphologic characteristics of positive LNs, including nodal grouping (2), extranodal extension (4), more than five nodal involvements (5), and nodal matting (6) are predominant independent prognostic factors for NPC patients’ survival. Moreover, the gross tumor volume of LNs not only predicted overall survival in NPC patients effectively but was also an indicator of the timely adjustment of therapeutic strategies for NPC patients, especially for those completing induction chemotherapy (IC) (7).

The current standard of care for locoregionally advanced NPC is cisplatin-based induction chemotherapy, followed by concurrent radiochemotherapy, which can yield excellent outcomes due to the early eradication of micrometastases (1, 8–10). Early identification of non-responders is beneficial for preventing excessive chemotherapy-related toxicities, improving and customizing treatment regimens for people; hence, precisely measuring and predicting response to IC appears to be essential.

In addition to morphologic imaging, advancements in functional imaging modalities have led to improving the ability to predict the primary tumor’s response to the treatment (11–15). Diffusion-weighted imaging (DWI) has been proven to be a valuable technique to accurately predict therapeutic response to IC (11, 12, 16). By providing more information about the underlying microstructure, diffusion kurtosis imaging (DKI) can be used to measure the therapeutic effect of IC (12–14, 16, 17). Intravoxel incoherent motion (IVIM) has proven its ability to assess and predict chemoradiotherapeutic response by quantifying and discriminating pure water molecular diffusion and the microcirculatory perfusion of the tissue (11, 18–22). Dynamic contrast-enhanced magnetic resonance imaging (DCE-MRI) is considered as a valuable tool for reflecting tumor angiogenesis density, vascular permeability, and tumor neoangiogenesis blood flow (23) and plays a distinct role in predicting efficacy, survival, and prognosis (15, 24, 25).

Several publications have explored the effectiveness of single fMRI techniques in assessing and predicting the effects of chemotherapy in malignancies. However, most of these investigations, to our knowledge, have mainly focused on primary tumors rather than metastatic LNs (11, 20). Furthermore, there has been no research comparing the efficacy of conventional DWI, DKI, IVIM, and DCE-MRI in predicting response to IC for the LNs yet. Therefore, the study was aimed to combined DWI, DKI, IVIM, and DCE-MRI techniques to identify and compare the value of fMRI parameters in predicting the early therapeutic response of LNs from NPC for the first time.

## Materials and methods

### Patient population and induction chemotherapy regimens

Our institution’s Institutional Review Board authorized the prospective single-center trial protocol (Clinical Trial Registration number: ChiCTR2100042863), and all participants signed written informed consent. Patients with pathologically confirmed NPC and at least one metastatic LN (according to the AJCC 8th Head and Neck Tumor Staging Criteria: TxN1-3M0) were prospectively included from January to May 2021. Each patient had two Magnetic resonance (MR) scans, one 3 days before the first IC cycle and the other 21–24 days after the second IC cycle. The inclusion criteria of the study were as follows: (1) The enlarged LNs with increased metabolic activity on ^18^F-FDG PET-CT scans (nodal SUV_max_ >2.5) performed within 1 week of the MRI scans were identified as metastatic LNs (24); (2) with nodal grouping, extranodal extension, and/or nodal matting; and (3) cervical LNs with a short-axis diameter of at least 15 mm; the lateral retropharyngeal nodes (RPNs) were considered metastatic if their shortest axial diameter was 10 mm, and any visible node in the median retropharyngeal group was considered malignant. There should be no previous malignancies or anticancer therapy for any of the individuals. There were no contraindications to IC, no MRI contraindications, and no metal implants in the mouth that may cause poor picture quality, which limited further investigation. All participants received two cycles of IC, docetaxel (70 mg/m^2^ on day 1) or paclitaxel-albumin (260 mg/m^2^ on day 1), and cisplatin (P) 40 mg/m^2^ on days 1 and 2.

### Functional MRI techniques

All MRI exams were performed on a 3.0 T MR scanner (Signa HDx, GE Healthcare, Milwaukee, WI, USA). The MRI protocols consisted of conventional DWI, DKI, IVIM, and DCE-MRI sequences. With a 16-channel neurovascular head and neck array coil, standard MRI sequences such as axial, sagittal, and coronal T2-weighted 2D turbo spin-echo pictures were acquired prior to DWI images. Then, using a single-shot echo-planar imaging sequence with b values of 0 and 600 mm^2^/s, axial DWI was obtained. A single-shot spin echo-planar imaging sequence with fast suppression was used for the axial DKI sequence. In addition, the diffusion gradients were applied in three orthogonal gradient diffusion directions. The IVIM was acquired using a prototyped integrated slice-specific dynamic Shim (iShim) sequence, the parameters of which were identical to those for DKI, except for the multiple b values (detailed information was summarized in **Supplemental File 1**). After the intravenous injection of 0.2 ml/kg of the contrast agent, DCE-MRI was acquired utilizing the FLASH 3D gradient-echo sequence (26) to generate four series of unenhanced pictures and 31 series of enhanced images without any delay (Gd-TPA, Magnevist; Bayer Schering, Berlin, Germany). The contrast agent was injected using an automated syringe pump at a rate of 2 ml/s, followed by a 20-ml saline flush at the same rate. DWI, DKI, IVIM, and DCE-MRI took 2:00, 4:09, 2:44, and 4:44 min to acquire, respectively. After that, in the same position as the T2WI, the axial, sagittal, and coronal postcontrast T1-weighted 2D turbo spin-echo pictures with fat saturation were taken (Detailed information was summarized in **Supplemental File 1**).

### Image analysis

The independent Linux workstation (Advantage Workstation version 4.6; GE Healthcare, US) was used to process the data. Pixel-wise apparent diffusion coefficient (ADC) maps were calculated by a two-variable linear least-square method based on a monoexponential model, using the following equation (27): *S*_*i*_=*S*_0_×*exp*(−*b*_*i*_×*ADC*). S_i_ means the MRI signal intensity at the diffusion weighting b_i_, while S_0_ represents that of non-diffusion weighted.

The DKI parameter maps were obtained using the postprocessed Functool software. In comparison with the monoexponential equation, the DKI model yielded two variables while S_0_ is known, according to the following equation (27, 28): $S_{i}=S_{0}\times \text{exp}\left(-b_{i}\times D+\frac{1}{6}bi^{2}\times D^{2}\times K\right)$ . with S_0_, D, and K as fitting variables, where S_i_ is the signal at a particular b value and S_0_ is the baseline signal without diffusion gradient (17). Accordingly, D is diffusivity, K describes the peakedness of a probability of water distribution (27, 28). The parameter MD represents the diffusion coefficient after adjusting for the non-Gaussian impact in normal diffusion, whereas MK represents non-Gaussian diffusion behavior. MK, as a non-Gaussian component, may show diffusion inhomogeneity that is hard to assess with standard DWI.

After the IVIM raw data were transferred to the Linux workstation, the parameter maps including D_slow_, D_fast_, and PF were calculated by MADC prototype software in the Functool software package. The assumption of the IVIM model was based on the translation movements at voxel levels. IVIM signal attenuation is the sum of the tissue and blood component, taking the shape of biexponential decay (22): *S*_*i*_/*S*_0_=(1−*PF*)×*exp*(−*b*×*D*_*slow*_)+*exp*(−*b*_*i*_×*D*_*slow*_+*D*_*fast*_) S_i_ means the signal intensity (SI) with diffusion gradient b_i_; S_0_ represented SI with the diffusion gradient being 0. D_slow_ means the true diffusion representing pure molecular diffusion (mm^2^/s), while D_fast_ represents the pseudo-diffusion coefficient as reflected by perfusion relative diffusion or incoherent microcirculation (mm^2^/s); PF acted as fractional perfusion related to microcirculation (22).

**Figure 1:**
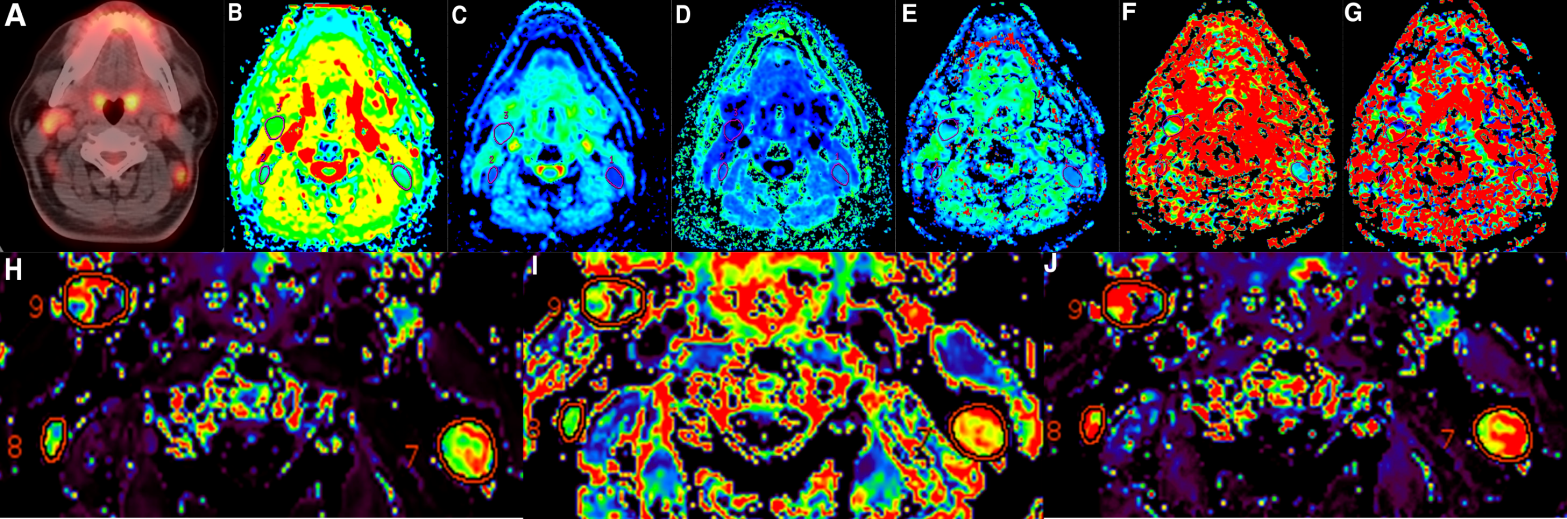
A 34-year-old man diagnosed with nasopharyngeal carcinoma has underwent PET-CT and multiparameter functional MR examinations. **(A)** PET/CT before induction chemotherapy (IC) confirmed the metastatic lymph nodes located in IIa and IIb, **(B)** apparent diffusion coefficient (ADC), **(C)** mean diffusion coefficient (MD), **(D)** mean kurtosis (MK), **(E)** pure molecular diffusion coefficient (D_slow_), **(F)** pseudo-diffusion coefficient (D_fast_), **(G)** perfusion fraction (PF), **(H)** volume transfer constant (K^trans^), **(I)** volume fraction of extravascular extracellular space (V_e_), and **(J)** the reflux rate constant between extravascular–extracellular space and plasma (K_ep_) maps derived from functional MRI (fMFRI) before induction chemotherapy (IC).

The DCE parameter maps, including K^trans^, V_e_, and K_ep_, were calculated using a two-compartment model that regards the tissue and plasma as two compartments. The transport was determined by the volume transfer constant: K^trans^ [from the blood plasma to the extracellular–extravascular space (EES)] and K_ep_ (from the EES to the blood plasma). The parameter V_e_ was defined as the EES fractional volume and was calculated by the following equation: *V*_*e*_=*K* ^*trans*^/*K*_*ep*_ All of the MR images were independently examined by two experienced radiologists who were blind to the patient’s clinical data. On fMRI parameter maps, the LNs’ region of interest was manually drawn with the goal of encompassing as much of the area as possible while avoiding the necrotic region (as shown in **Figure 1**). For example, in DWI with b = 600 s/mm^2^, we chose the largest cross-sectional area of LNs to delineate ROIs on the basis of morphological T2-weighted images and/or postcontrast T1-weighted images. As for DKI and IVIM, the method was the same as DWI, and once the ROIs were drowned on the specific b-value image, it would be automatically duplicated to the other parameter maps. The amount of time spent analyzing each sequence ranged from 3 to 5 min. Overall, ADC values took the least amount of time to compute, while DCE-MRI postprocessing took the most time. The final results were recorded as the mean value of two observers’ measurements. The tumor volume was calculated as follows: *volume*=*area* *of* *leision*×(*thickness* *of* *slice*+*interslice* *gap*) The following equation was used to compute the changes in the parameters before and after IC:


Δ(parameters)%=(post(parameters)−pre(parameters))/pre(parameters)%

### Response evaluation

The metastatic LNs from NPC patients were identified as the only target lesion to be evaluated, while the primary tumor was not taken into consideration. Separating RLNs from the primary tumor allowed them to be identified. A contrast-enhancing ring or a difference in signal intensity compared to the primary tumor clearly indicated the LNs that were contiguous with the primary tumor. The target lesions were classified as the responding group (RG) and non-responding group (NRG) after two cycles of chemotherapy according to the criteria of Response Evaluation Criteria in Solid Tumors (RECIST Version1.1) (29). RG includes patients with complete response (disappearance of the metastatic LN) and partial response (at least a 30% decrease in the diameter of the LN, taking as reference the baseline diameter). NRG includes progressive disease (at least a 20% increase in the diameter of the LN, taking as reference the baseline diameter), and stable disease (<30% decrease and <20% increase in the diameter of the LN). T2WI was used to compute the volume of LNs. The regression ratio and tumor volume reduction ratio were calculated as the following equation:


Δ(diameter or volume)=post(diameter or volume)−pre(diameter or volume)

Δ(diameter or volume)%=Δ(diameter or volume)/pre(diameter or volume)%

### Statistical analysis

The chi-square test, adjustment for continuity, or Fisher’s exact test were used to compare categorical data. The mean value of these parameters was compared before and after treatment using the paired t-test or Wilcoxon rank-sum test (according to the normality of data distributed). An independent-sample t-test or Mann–Whitney U test (according to the normality of data distributed) was performed to compare the mean value of the parameters and changes of parameters after treatment between the RG and the NRG. The significant parameters are fitted by logistic regression analysis to produce a new predictive factor (PRE). The receiver operating characteristic (ROC) was used to identify and compare the efficacy of the significant parameters in predicting IC outcomes. The intraobserver reproducibility of parameters was analyzed using the intraclass correlation coefficient (ICC). Statistical analyses were performed in R software (version 4.1.0; http://www.r-project.org). Statistical significance was defined as a p-value of less than 0.05.

## Results

### Participants and metastatic lymph node characteristics

The study included 94 LNs from 40 persons, including 8 men and 32 women, with an average age of 43.33 ± 13.79 years (range from 18 to 67). A total of 52 LNs were allocated to the RG after two cycles of IC, while the remaining 42 were classified as NRG. The tumor volume decrease rate in the RG (76.85 ± 12.89%) was much higher than in the NRG (30.99 ± 17.55%). Between the RG and the NRG, there was no significant difference in the T stage, pathological categorization, chemotherapeutic regimens, or volume-pre (**Table 1**). The intrareproducibility of the metrics’ values was excellent, and the ICC of ADC-pre, ADC-post, MD-pre, MD-post, MK-pre, MK-post, D_slow_-pre, D_slow_ -post, D_fast_-pre, D_fast_ -post, PF-pre, PF-post, K^trans^-pre, K^trans^-post, K_ep_-pre, K_ep_-post, V_e_-pre, and V_e_-post were 0.855, 0.91, 0.889, 0.924, 0.839, 0.855, 0.896, 0.880, 0.866,0.856, 0.915, 0.831, 0.847, 0.893, 0.895, 0.878, 0.903, and 0.863, respectively.

**Table 1:** Clinical characteristics of lymph nodes in the responding group (RG) and the non-responding group (NRG).

	RG (n = 52)	NRG (n = 42)	t/χ^2^	*p*
**AJCC T classification**			3.06	0.379
T1	0 (0%)	1 (2.4%)		
T2	30 (57.7%)	21 (50.0%)		
T3	16 (30.8%)	11 (26.2%)		
T4	6 (11.5%)	9 (21.4%)		
**Pathological classification**			1.68	0.433
Non-cornification undifferentiated	25 (48.1%)	24 (57.1%)		
Non-cornification differentiated	27 (51.9%)	18 (42.9%)		
**IC regimes**			1.13	0.296
Docetaxel + cisplatin	42 (80.8%)	30 (71.4%)		
Paclitaxel-albumin + cisplatin	10 (19.2%)	12 (28.6%)		
**V–pre (cm^3^)**	12.26 ± 9.16	9.31 ± 10.09	1.48	0.142
**V–post (cm^3^)**	2.95 ± 3.05	6.60 ± 6.74	-3.49	**0.001**
**ΔV %**	76.85 ± 12.89	30.99 ± 17.55	14.60	**0.000**

Data represent the number of patients, and the data in parentheses are percentages. IC, induction chemotherapy; RG, responding group; NRG, non-responding group; V, volume of LNs.

The bold values represent these parameters showed a significant differences between the groups and p < 0.05.

### Comparison of functional MRI parameters before and after induction chemotherapy

**Figure 2:**
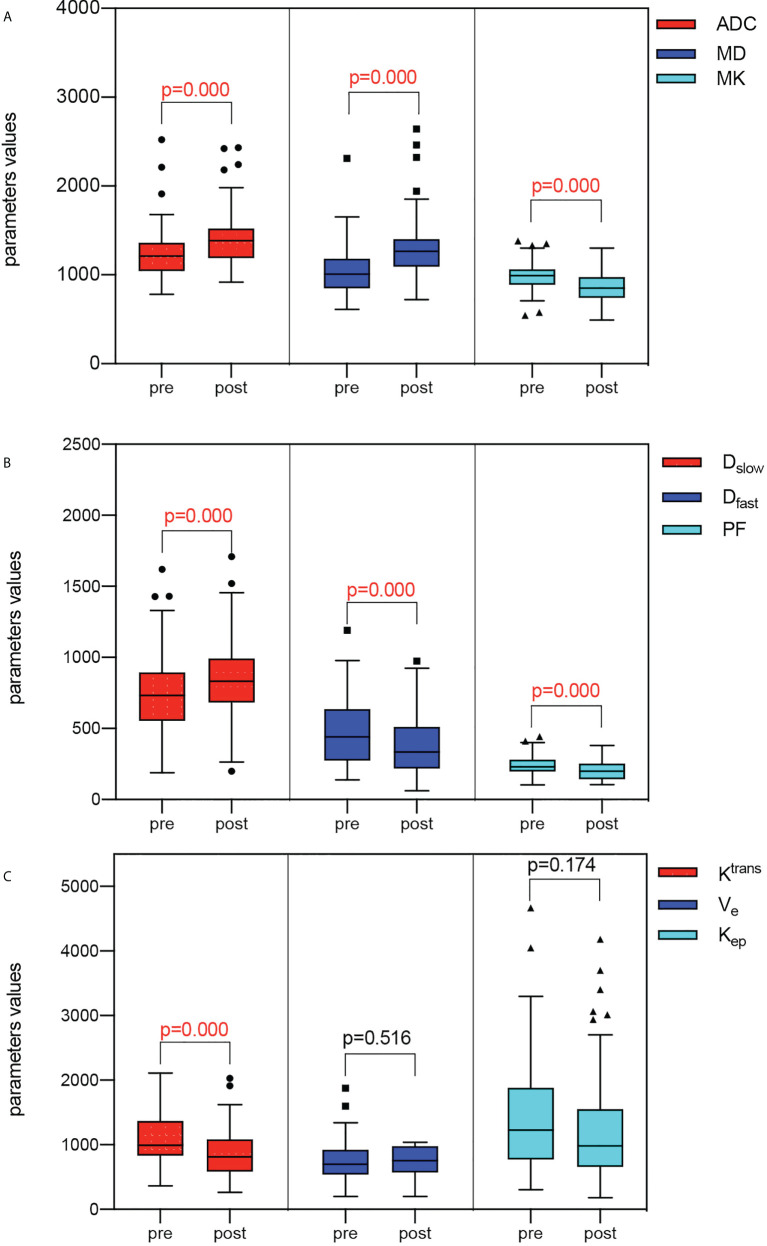
Box and whisker plot of the fMRI parameters before and after IC. **(A)** Comparisons of ADC, MD, and MK before and after IC showed that ADC and MD increased while MK decreased after IC. **(B)** Comparisons of D_slow_, D_fast_, and PF before and after IC showed that D_slow_ increased while D_fast_ and PF decreased after IC. **(C)** Comparisons of K^trans^, K_ep_, and V_e_ before and after IC showed that K^trans^ decreased after IC, while there were no differences in K_ep_ and V_e_.

Except for V_e_ and K_ep_, there were statistically significant variations in ADC, MD, MK, D_slow_, D_fast_, PF, and K^trans^ before and after IC. After two cycles of IC, the mean value of ADC, MD, D_slow_ significantly increased, while MK, D_fast_, PF, and K^trans^ values decrease dramatically (**Table 2**; **Figure 2**).

**Table 2:** The comparison of functional MRI (fMRI) parameters before and after induction chemotherapy.

	Pre-IC	Post-IC	*t/Z*	*p*
**ADC (×10^-6^ mm^2^/s)**	1,232.62 ± 281.61	1,394.00 ± 291.55	-4.53	**0.000**
**MD (×10^-6^ mm^2^/s)**	1,052.05 ± 263.43	1,286.16 ± 320.15	-7.04	**0.000**
**MK (×10^-6^)**	994.97 ± 164.62	852.36 ± 171.83	-7.50	**0.000**
**D_slow_ (×10^-6^ mm^2^/s)**	727.73 ± 251.39	911.93 ± 611.94	-2.59	**0.011**
**D_fast_ (×10^-4^ mm^2^/s)**	474.37 ± 233.04	380.43 ± 224.13	3.19	**0.002**
**PF (×10^-4^)**	236.09 ± 71.30	199.22 ± 62.69	6.47	**0.000**
**K^trans^ (×10^-3^/min)**	1,065.80 ± 352.86	853.58 ± 387.82	7.20	**0.000**
**V_e_ (×10^-3^)**	711.51 ± 274.07	739.17 ± 230.93	-0.65	0.516
**K_ep_ (×10^-3^/min)**	1,366.72 ± 833.90	1,225.94 ± 816.52	1.37	0.174

IC, induction chemotherapy; ADC, apparent diffusion coefficient (×10^-6^mm^2^/s); MD, mean diffusion (×10^-6^mm^2^/s); MK, mean kurtosis (×10^-6^); D_slow_, true diffusion coefficient (×10^-6^ mm^2^/s); D_fast_, pseudo-diffusion coefficient (×10^-4^ mm^2^/s); PF, perfusion fraction (×10^-4^); K^trans^, volume transfer constant (×10^-3^/min); Ve, extracellular extravascular space (×10^-3^); K_ep_, rate constant (×10^-3^/min).

The bold values represent these parameters showed a significant differences between the groups and p < 0.05.

### Comparisons of functional MRI parameters between responding group and non-responding group

**Figure 3:**
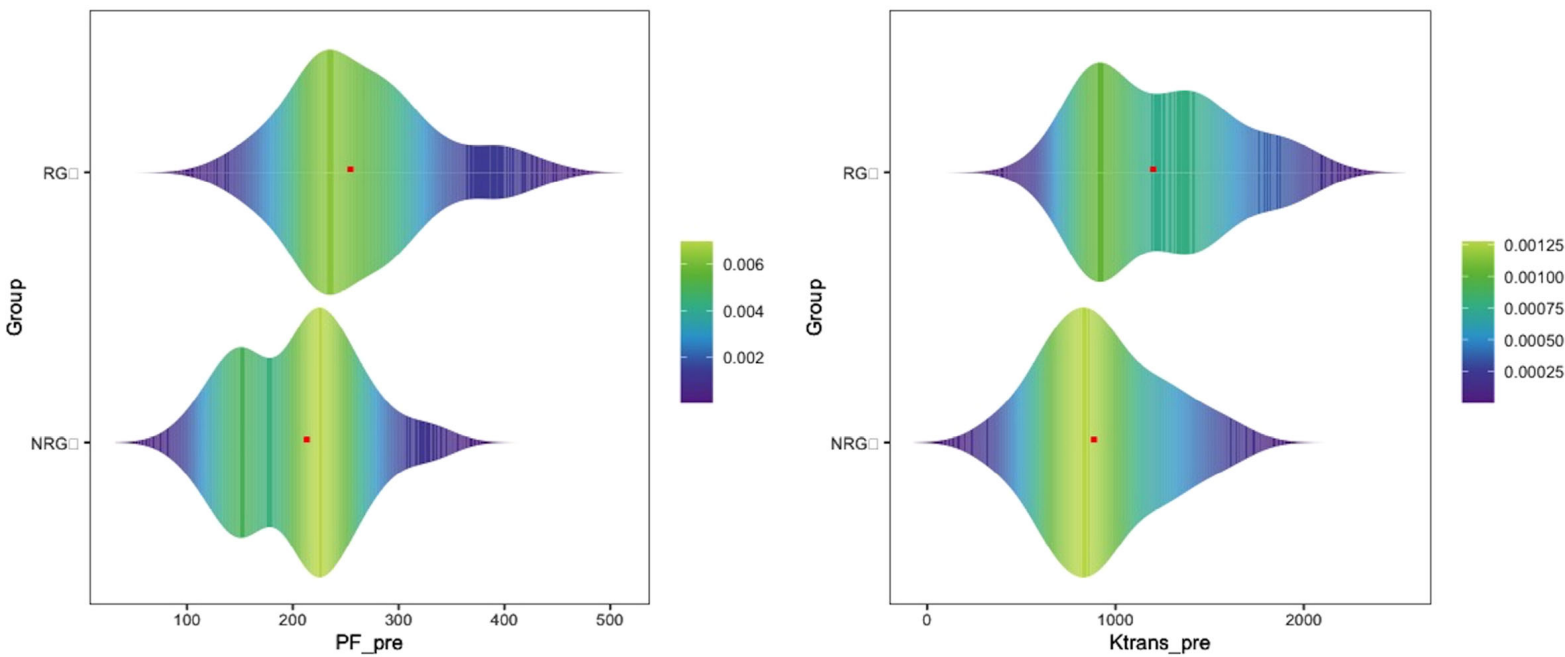
Violin distribution and density of PF-pre and K^trans^-pre values in the responding group (RG) and non-responding group (NRG). The red dot represents the median PF-pre and K^trans^-pre values for the two groups. Colors in the plot are correlated with the sample density. The value of PF and K^trans^ was higher in RG than that in the NRG.

Statistically significant differences were identified in PF-pre, PF-post, ΔPF%, K^trans^-pre, and K^trans^-post between the RG and the NRG. The mean value of ΔADC% in the RG showed an upward trend, while MK-post values showed a decreasing trend, but there were no significant differences compared with the NRG (*p>0.05*). However, the other parameters derived from fMRI showed no significant differences between groups (**Table 3**; **Figure 3**).

**Table 3:** The fMRI parameters with statistical differences between the RG and the NRG.

	RG (n = 52)	NRG (n = 42)	*t/U*	*p*
**ADC-pre (×10^-6^ mm^2^/s)**	1,251.65 ± 210.64	1,202.02 ± 210.64	0.87	0.383
**ADC-post (×10^-6^ mm^2^/s)**	1,375.59 ± 235.54	1413.29 ± 342.48	-0.60	0.552
**MD-pre (×10^-6^ mm^2^/s)**	1,024.44 ± 202.53	1066.17 ± 318.57	-0.77	0.442
**MD-post (×10^-6^ mm^2^/s)**	1,263.82 ± 228.33	1309.57 ± 395.87	-0.66	0.511
**MK-pre (×10^-6^)**	993.17 ± 151.61	981.02 ± 173.83	0.36	0.718
**MK-post (×10^-6^)**	883.30 ± 151.83	819.95 ± 186.90	1.73	0.088
**D_slow_-pre (×10^-6^ mm^2^/s)**	741.79 ± 261.22	762.76 ± 284.00	-0.37	0.711
**D_slow_-post (×10^-6^ mm^2^/s)**	828.68 ± 273.48	999.14 ± 826.25	-1.30	0.198
**D_fast_-pre (×10^-4^ mm^2^/s)**	507.27 ± 243.43	431.76 ± 216.35	1.57	0.120
**D_fast_-post (×10^-4^ mm^2^/s)**	393.50 ± 239.72	366.74 ± 208.56	0.55	0.583
**PF-pre (×10^-4^)**	263.71 ± 68.44	205.60 ± 56.83	4.41	**0.000**
**PF-post (×10^-4^)**	214.25 ± 66.55	183.48 ± 54.83	2.33	**0.022**
**K^trans^-pre (×10^-3^/min)**	1,226.75 ± 385.23	925.81 ± 311.04	4.10	**0.000**
**K^trans^-post (×10^-3^/min)**	940.64 ± 369.19	762.38 ± 390.16	2.18	**0.032**
**V_e_-pre (×10^-3^)**	740.02 ± 321.48	706.95 ± 221.05	0.57	0.572
**V_e_-post (×10^-3^)**	733.50 ± 243.99	745.12 ± 219.22	-2.32	0.817
**K_ep_-pre (×10^-3^/min)**	1,398.35 ± 707.56	1426.62 ± 961.53	-0.16	0.870
**K_ep_-post (×10^-3^/min)**	1,104.55 ± 635.26	1353.12 ± 962.58	-1.42	0.159
**ΔADC%**	11.13 ± 20.68	21.073 ± 31.37	-1.86	0.066
**ΔMD%**	25.77 ± 32.31	25.88 ± 29.05	-0.02	0.987
**ΔMK%**	-10.87 ± 16.65	-15.74 ± 15.35	1.41	0.163
**ΔD_slow_%**	-29.18 ± 54.17	-55.40 ± 191.19	0.87	0.385
**ΔD_fast_%**	6.56 ± 96.23	-2.81 ± 71.99	0.51	0.612
**ΔPF%**	19.05 ± 14.85	6.48 ± 30.80	2.43	**0.017**
**ΔK^trans^%**	20.44 ± 24.82	20.04 ± 20.14	0.08	0.935
**Δ V_e_%**	-25.35 ± 70.73	-21.03 ± 64.36	-0.29	0.768
**Δ K_ep_ %**	-4.40 ± 66.75	-20.83 ± 107.05	0.86	0.393

V, volume of tumor (cm^3^); ADC, apparent diffusion coefficient (×10^-6^mm^2^/s); MD, mean diffusion (×10^-6^mm^2^/s); MK, mean kurtosis (×10^-6^); D_slow_, true diffusion coefficient (×10^-6^mm^2^/s). Data were reported as mean values ± standard error.

The bold values represent these parameters showed a significant differences between the groups and p < 0.05.

### The diagnostic performance of MRI parameters

**Figure 4:**
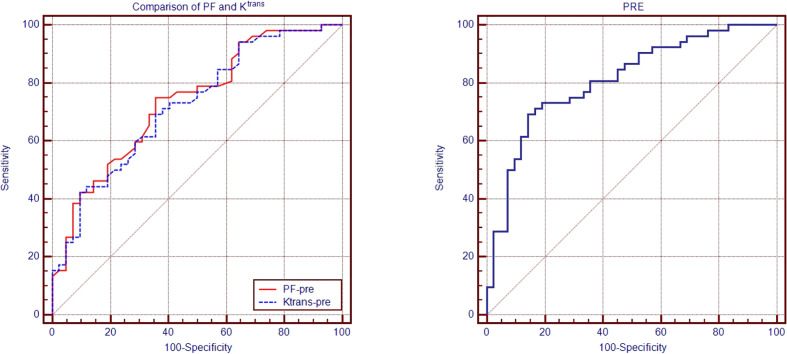
The receiver operating characteristic ROC of K^trans^, PF, and PRE in predicting response to IC. The areas under the ROC curves for PF-pre, K^trans^ -pre, and PRE were 0.736, 0.722, and 0.810, respectively.

The parameters PF and K^trans^ were fitted by logistic regression analysis to produce a new PRE. the calculation formula was as follows: *Logit*(*P*)=−5.654+0.013∗*PF*+0.003∗*K* ^*trans*^. $PRE=\frac{e^{Logit\left(P\right)}}{1+e^{Logit\left(P\right)}}$ The areas under the ROC curves for PF-pre, K^trans^ -pre, and PRE were 0.736, 0.722, and 0.810, respectively, with the optimal cutoff values of 222×10^-4^, 934×10^-3^/min, and 0.6624, respectively (**Table 4**; **Figure 4**). However, there were no statistically significant differences between the other parameters.

**Table 4:** Diagnostic efficacy of K^trans^-pre, PF-pre, and PRE.

	AUC	95%CI	Youden	Cutoff	sensitivity	specificity	+LR	-LR	+PV	-PV
**PF-pre (×10^-4^)**	0.736	(0.635,0.822)	0.393	222	75.00%	64.29%	2.10	0.39	72.2	67.5
**K^trans^-pre (×10^-3^/min)**	0.722	(0.620,0.809)	0.335	934	69.23%	64.29%	1.94	0.48	70.6	62.8
**PRE**	0.810	(0.715,0.883)	0.549	0.6224	69.23%	85.71%	4.85	0.36	85.7	69.2

AUC, area under the curve; 95% CI, 95% confidence interval; +LR, positive likelihood ratio; -LR, negative likelihood ratio; +PV, positive predictive value; -PV, negative predictive value.

## Discussion

The present study was designed to analyze and compare the utility of pretreatment fMRI parameters in predicting therapeutic response to IC in NPC metastatic LNs. Except for V_e_ and K_ep_, all of the parameters generated from fMRI could reflect changes in the tissue microstructure following IC. After IC, the mean values of ADC, MD, and D_slow_ increased dramatically, but MK, D_fast_, PF, and K^trans^ values dropped. Meanwhile, the RG had much higher PF-pre and K^trans^-pre values than the NRG. Furthermore, ROC analysis revealed that fMRI parameters have the potential to distinguish the RG from the NRG using the indications of PF-pre, K^trans^-pre, and PRE. The accurate prediction of efficacy before the treatment of cervical LNs in patients with NPC can improve both their treatment and prognosis. To reduce the local regional recurrence rate, the dose of radiation can be raised suitably for patients with poor IC effectiveness. It is particularly important for individuals who are going to receive concurrent chemoradiotherapy only.

Regardless of the fact that ADC is an “apparent” metric with no direct biophysical basis, it is believed to be linked to the extracellular space, which is determined by tissue architectural elements (28). The utility of DWI in monitoring therapy response has been proven in a variety of cancers during the last few decades. ADC had a lot of promise as an imaging biomarker for predicting early treatment response (12). Our findings revealed that ADC values increased significantly after IC, possibly because of chemotherapy-induced structural necrosis and decreased cellularity in the tumor. In addition, these results were consistent with the previous findings (13, 19, 30, 31). There was no statistically significant difference regarding ADC-pre between the RG and the NRG. This was disparate from previous conclusions of primary tumor (31), Marzi et al. also revealed that patients with regional control showed significantly lower pretreatment ADC values compared to regional failure patients. However, it was consistent with results of LNs from NPC (11), Lu et al. found that the initial ADC showed no significant difference between nodes with a PR or CR.

DKI provides an opportunity to get further insights into the actual diffusion of water molecules *in vivo* (28). In addition to MD, the diffusion kurtosis coefficient (MK) has been introduced to enable the evaluation of non-Gaussian diffusion behavior and quantitative analysis of the deviation’s magnitude. After IC, we saw an increase in the MD values and a decrease in the MK values of LNs in our study. The mechanism for increased MD values was the same as for ADC, whereas the decline in MK values could be explained by the extracellular space expanding faster and becoming more isotropic (32). The results have also been verified in the primary tumor (13), but there have been no specific studies focused on the predictive efficacy in LNs from NPC before. According to our findings, the metrics generated from DKI revealed no significant changes between the two groups, but MK-post values of LNs in the RG exhibited a declining tendency. The negative finding of LNs differed from that of the primary tumor of NPC (13). The explanation for this might be that the metastatic LNs were highly homogenous, resulting in similar cellularity density compared with the relatively heterogeneous primary tumors extension.

IVIM reflects the random microscopic motion of water molecules in the intracellular, extracellular space and microcirculation of blood (33). IVIM can simultaneously obtain the information of tumor tissue diffusion and perfusion and may serve as predictors of effective response (18). We found that D_slow_ values increased whereas D_fast_ and PF values decreased significantly after IC. In addition, this could be explained by a dramatical decrease in the cellularity and microvessels, as D_slow_ reflects the true water diffusion and was related to extracellular spaces. However, the present study failed to detect the difference in D_slow_ between the RG and the NRG, which was inconsistent with previous results. Marzi et al. found that patients with RC showed significantly lower pretreatment D_slow_ values compared to RF patients (20). The differences of the primary outcomes identified by the study may be the main reason for this discrepancy. PF indicates the fraction of perfusion-related microvessels in the total tissue to some extent. The results also showed that the PF values in the RG were greater than in the NRG, which was in line with the previous findings (11). They found that the mean initial PF value was significantly higher in patients with a PR relative to patients with a CR. It was speculated that the presence of more abundant microvessels in the metastatic LNs of RG.

DCE-MRI allows for probing perfusion and microvessel permeability using the Tofts pharmacokinetic analysis of images. As known, K^trans^, K_ep_, and their ratio V_e_ can be used to quantitative analyze the physiological properties of tumor (34). K^trans^ was the most important indicator, representing the blood volume, vascular endothelial permeability, and surface area of microvessels in tumor tissues, and reflecting the volume transfer constant (from blood plasma to the EES). Our findings also revealed that following IC, K^trans^ values decrease considerably, although Kep and Ve showed no significant changes. Reduced microvascularity in LNs could explain the decrease in K^trans^ after treatment. We also discovered that K^trans^ values in the RG differed significantly from the NRG, which was in line with previous findings. It was reported that K^trans^ was a potential marker of predicting response right after one IC cycle for NPC patients (35). Especially, pretreatment primary lesions quantitative DCE-MRI may be valuable in predicting the prognosis for NPC (36). It is not hard to follow actually; higher K^trans^ values mean higher permeability and perfusion, which can contribute to the improved ability of chemotherapeutic drugs delivered to the tissue (37). As mentioned above, both D_fast_ and K^trans^ are thought to be related to tissue perfusion, and the change trend of D_fast_ before and after IC was the same as K^trans^. Furthermore, K^trans^ in the RG was higher than that in NRG, which is consistent with D_fast_, although D_fast_ showed no significant difference between the two groups. Perfusion-related parameters including PF and K^trans^ have a great potential to predict therapy response.

To our knowledge, the study presented a first attempt to identify and compare the value of fMRI metrics in predicting therapeutic response to IC in metastatic LNs in NPC. We found that the best performance of pretreatment metrics to discriminate responders from non-responders was K^trans^ and PF. The attempts to combine multiple statistically significant MRI parameters succussed to generate a stronger predictor PRE that was fitted by PF and K^trans^ using the way of the input of logistic regression analysis. PF, K^trans^, and PRE showed a promising value of predicting response to IC, with an area under the ROC of 736, 0.722, and 0.810 respectively. These results were partly in line with those of Wong et al. (11, 38), although the other parameters seemed to be not a powerful predictor of response to IC. Lu et al. (11) reported that the AUC of the pretreatment PF for distinguishing the complete response group from the partial response group was 0.920. The sensitivity and specificity of PF in predicting treatment response to chemoradiotherapy were 86.7% and 100%, respectively.

There are several limitations to this study that must be acknowledged. First and foremost, there is the single-center study, which has intrinsic flaws. Second, due to time constraints, the cervical metastatic LNs scanned and evaluated in the study were mostly situated in level II, III, and retropharyngeal space, with the exception of level IV. It is unclear whether the lack of data from metastatic LNs in level IV influenced the final results. Thirdly, the pathological outcomes should be compared to the responses of metastatic LNs to IC. Unfortunately, surgery is not a recommended treatment for NPC, and the biopsies of a large number of regional LNs are similarly impractical. Finally, chemosensitivity cannot directly represent radiosensitivity. Identifying patients with high sensitivity to IC who could potentially achieve complete response or partial response and offering them standard (or intensified) neoadjuvant chemotherapy could therefore improve long-term survival. Further study is warranted and the adiomics of fMRI parameter-maps for detecting microstructural changes and predicting treatment response is a potential issue in this respect.

## Conclusions

The purpose of the current study was to identify and compare the efficacy of conventional DWI, DKI, IVIM, and DCE-MRI in predicting early response to IC in metastatic LNs from NPC. The pretreatment K^trans^ and PF values emerged as the reliable PREs of therapeutic response, which demonstrated that fMRI perfusion–related parameters have the potential to early predict the efficacy of IC in metastatic LNs from NPC patients. Multicenter radiomics trials are urgently needed to verify our findings.

## Data availability statement

The original contributions presented in the study are included in the article/**Supplementary Material**. Further inquiries can be directed to the corresponding authors.

## Ethics statement

The studies involving human participants were reviewed and approved by the ethics board of the Chinese PLA General Hospital. Written informed consent to participate in this study was provided by the participants’ legal guardian/next of kin.

## Author contributions

DZ, XF, WF, LM, BNC: design of the study, interpretation of data, draft of work. DZ, XF, WF, LM, BNC, LLM, YL, NC, JL, XZ, ML, XG, BYC, CW, XT: Data curation, interpretation of data, draft of work. All authors contributed to the article and approved the submitted version.

## Conflict of interest

The authors declare that the research was conducted in the absence of any commercial or financial relationships that could be construed as a potential conflict of interest.

## Publisher’s note

All claims expressed in this article are solely those of the authors and do not necessarily represent those of their affiliated organizations, or those of the publisher, the editors and the reviewers. Any product that may be evaluated in this article, or claim that may be made by its manufacturer, is not guaranteed or endorsed by the publisher.

## Abbreviations

fMRI, functional magnetic resonance imaging; IC, induction chemotherapy; LNs, lymph nodes; NPC, nasopharyngeal carcinoma; DKI, diffusion kurtosis imaging; IVIM, intravoxel incoherent motion; DCE-MRI, dynamic contrast-enhanced magnetic resonance imaging; ADC, apparent diffusion coefficient; MD, mean diffusivity; MK, mean kurtosis; D_slow_, pure molecular diffusion coefficient; D_fast_, pseudo-diffusion coefficient; PF, fractional perfusion; K^trans^, volume transfer constant; K_ep_, reflux rate constant between extravascular–extracellular space and plasma; V_e_, volume fraction of extravascular extracellular space; RG, responding group; NRG, non-responding group.

## References

[1] Chen Y-P Chan ATC Le Q-T Blanchard P Sun Y Ma J . Nasopharyngeal carcinoma. Lancet (2019) 394(10192):64–80. doi: 10.1016/s0140-6736(19)30956-0 31178151

[2] Liu Y Chen S Dong A Ai F Quan T Cui C . Nodal grouping in nasopharyngeal carcinoma: Prognostic significance, n classification, and a marker for the identification of candidates for induction chemotherapy. Eur Radiol (2020) 30(4):2115–24. doi: 10.1007/s00330-019-06537-6 31811429

[3] Ma H Liang S Cui C Zhang Y Xie F Zhou J . Prognostic significance of quantitative metastatic lymph node burden on magnetic resonance imaging in nasopharyngeal carcinoma: A retrospective study of 1224 patients from two centers. Radiother Oncol (2020) 151:40–6. doi: 10.1016/j.radonc.2020.07.023 32679310

[4] Ai QY King AD Poon DMC Mo FKF Hui EP Tong M . Extranodal extension is a criterion for poor outcome in patients with metastatic nodes from cancer of the nasopharynx. Oral Oncol (2019) 88:124–30. doi: 10.1016/j.oraloncology.2018.11.007 30616782

[5] Dumrongpisutikul N Luangcharuthorn K . Imaging characteristics of nasopharyngeal carcinoma for predicting distant metastasis. Clin Radiol (2019) 74(10):818.e9–.e15. doi: 10.1016/j.crad.2019.06.031 31400806

[6] Ma H Qiu Y Li H Xie F Ruan G Liu L . Prognostic value of nodal matting on mri in nasopharyngeal carcinoma patients. J Magn Reson Imaging (2021) 53(1):152–64. doi: 10.1002/jmri.27339 32860315

[7] Li J-Y Huang C-L Luo W-J Zhang Y Tang L-L Peng H . An integrated model of the gross tumor volume of cervical lymph nodes and pretreatment plasma Epstein-Barr virus DNA predicts survival of nasopharyngeal carcinoma in the intensity-modulated radiotherapy era: A big-data intelligence platform-based analysis. Ther Adv Med Oncol (2019) 11:1758835919877729. doi: 10.1177/1758835919877729 31598143 PMC6763945

[8] Kong L Zhang Y Hu C Guo Y Lu JJ . Effects of induction docetaxel, platinum, and fluorouracil chemotherapy in patients with stage iii or Iva/B nasopharyngeal cancer treated with concurrent chemoradiation therapy: Final results of 2 parallel phase 2 clinical trials. Cancer (2017) 123(12):2258–67. doi: 10.1002/cncr.30566 28192641

[9] Liu LT Chen QY Tang LQ Guo SS Guo L Mo HY . Neoadjuvant or adjuvant chemotherapy plus concurrent crt versus concurrent crt alone in the treatment of nasopharyngeal carcinoma: A study based on ebv DNA. J Natl Compr Canc Netw (2019) 17(6):703–10. doi: 10.6004/jnccn.2018.7270 31200353

[10] Lv X Cao X Xia WX Liu KY Qiang MY Guo L . Induction chemotherapy with lobaplatin and fluorouracil versus cisplatin and fluorouracil followed by chemoradiotherapy in patients with stage iii-ivb nasopharyngeal carcinoma: An open-label, non-inferiority, randomised, controlled, phase 3 trial. Lancet Oncol (2021) 22(5):716–26. doi: 10.1016/S1470-2045(21)00075-9 33857411

[11] Lu L Li Y Li W . The role of intravoxel incoherent motion mri in predicting early treatment response to chemoradiation for metastatic lymph nodes in nasopharyngeal carcinoma. Adv Ther (2016) 33(7):1158–68. doi: 10.1007/s12325-016-0352-3 27294489

[12] Chung SR Choi YJ Suh CH Lee JH Baek JH . Diffusion-weighted magnetic resonance imaging for predicting response to chemoradiation therapy for head and neck squamous cell carcinoma: A systematic review. Korean J Radiol (2019) 20(4):649–61. doi: 10.3348/kjr.2018.0446 PMC6424826 30887747

[13] Chen Y Ren W Zheng D Zhong J Liu X Yue Q . Diffusion kurtosis imaging predicts neoadjuvant chemotherapy responses within 4 days in advanced nasopharyngeal carcinoma patients. J Magn Reson Imaging (2015) 42(5):1354–61. doi: 10.1002/jmri.24910 25873208

[14] Zheng D Lai G Chen Y Yue Q Liu X Chen X . Integrating dynamic contrast-enhanced magnetic resonance imaging and diffusion kurtosis imaging for neoadjuvant chemotherapy assessment of nasopharyngeal carcinoma. J Magn Reson Imaging (2018) 48(5):1208–16. doi: 10.1002/jmri.26164 29693765

[15] Paudyal R Chen L Oh JH Zakeri K Hatzoglou V Tsai CJ . Nongaussian intravoxel incoherent motion diffusion weighted and fast exchange regime dynamic contrast-Enhanced-Mri of nasopharyngeal carcinoma: Preliminary study for predicting locoregional failure. Cancers (Basel) (2021) 13(5):1128–42. doi: 10.3390/cancers13051128 PMC7961986 33800762

[16] Partridge SC Zhang Z Newitt DC Gibbs JE Chenevert TL Rosen MA . Diffusion-weighted mri findings predict pathologic response in neoadjuvant treatment of breast cancer: The acrin 6698 multicenter trial. Radiology (2018) 289(3):618–27. doi: 10.1148/radiol.2018180273 PMC6283325 30179110

[17] Zhu L Pan Z Ma Q Yang W Shi H Fu C . Diffusion kurtosis imaging study of rectal adenocarcinoma associated with histopathologic prognostic factors: Preliminary findings. Radiology (2017) 284(1):66–76. doi: 10.1148/radiol.2016160094 27929929

[18] Xiao Y Chen Y Chen Y He Z Yao Y Pan J . Longitudinal assessment of intravoxel incoherent motion diffusion weighted imaging in evaluating the radio-sensitivity of nasopharyngeal carcinoma treated with intensity-modulated radiation therapy. Cancer Res Treat (2019) 51(1):345–56. doi: 10.4143/crt.2018.089 PMC6334000 29764118

[19] Xiao-ping Y Jing H Fei-ping L Yin H Qiang L Lanlan W . Intravoxel incoherent motion mri for predicting early response to induction chemotherapy and chemoradiotherapy in patients with nasopharyngeal carcinoma. J Magn Reson Imaging (2016) 43(5):1179–90. doi: 10.1002/jmri.25075 26540374

[20] Marzi S Piludu F Sanguineti G Marucci L Farneti A Terrenato I . The prediction of the treatment response of cervical nodes using intravoxel incoherent motion diffusion-weighted imaging. Eur J Radiol (2017) 92:93–102. doi: 10.1016/j.ejrad.2017.05.002 28624026

[21] Song T Yao Q Qu J Zhang H Zhao Y Qin J . The value of intravoxel incoherent motion diffusion-weighted imaging in predicting the pathologic response to neoadjuvant chemotherapy in locally advanced esophageal squamous cell carcinoma. Eur Radiol (2021) 31(3):1391–400. doi: 10.1007/s00330-020-07248-z 32901300

[22] Szubert-Franczak AE Naduk-Ostrowska M Pasicz K Podgorska J Skrzynski W Cieszanowski A . Intravoxel incoherent motion magnetic resonance imaging: Basic principles and clinical applications. Pol J Radiol (2020) 85:e624–e35. doi: 10.5114/pjr.2020.101476 PMC7757509 33376564

[23] Syed AK Whisenant JG Barnes SL Sorace AG Yankeelov TE . Multiparametric analysis of longitudinal quantitative mri data to identify distinct tumor habitats in preclinical models of breast cancer. Cancers (Basel) (2020) 12(6):1682–1702. doi: 10.3390/cancers12061682 PMC7352623 32599906

[24] Huang B Kwong DL Lai V Chan Q Whitcher B Khong PL . Dynamic contrast-enhanced magnetic resonance imaging of regional nodal metastasis in nasopharyngeal carcinoma: Correlation with nodal staging. Contrast Media Mol Imaging (2017) 2017:4519653. doi: 10.1155/2017/4519653 29097922 PMC5612710

[25] Chan SC Yeh CH Chang JT Chang KP Wang JH Ng SH . Combing mri perfusion and (18)F-fdg Pet/Ct metabolic biomarkers helps predict survival in advanced nasopharyngeal carcinoma: A prospective multimodal imaging study. Cancers (Basel) (2021) 13(7):1550–64. doi: 10.3390/cancers13071550 PMC8036946 33800542

[26] Heethuis SE van Rossum PS Lips IM Goense L Voncken FE Reerink O . Dynamic contrast-enhanced mri for treatment response assessment in patients with oesophageal cancer receiving neoadjuvant chemoradiotherapy. Radiother Oncol (2016) 120(1):128–35. doi: 10.1016/j.radonc.2016.05.009 27296409

[27] Arab A Wojna-Pelczar A Khairnar A Szabo N Ruda-Kucerova J . Principles of diffusion kurtosis imaging and its role in early diagnosis of neurodegenerative disorders. Brain Res Bull (2018) 139:91–8. doi: 10.1016/j.brainresbull.2018.01.015 29378223

[28] Rosenkrantz AB Padhani AR Chenevert TL Koh DM De Keyzer F Taouli B . Body diffusion kurtosis imaging: Basic principles, applications, and considerations for clinical practice. J Magn Reson Imaging (2015) 42(5):1190–202. doi: 10.1002/jmri.24985 26119267

[29] Eisenhauer EA Therasse P Bogaerts J Schwartz LH Sargent D Ford R . New response evaluation criteria in solid tumours: Revised recist guideline (Version 1.1). Eur J Cancer (2009) 45(2):228–47. doi: 10.1016/j.ejca.2008.10.026 19097774

[30] King AD Chow KK Yu KH Mo FK Yeung DK Yuan J . Head and neck squamous cell carcinoma: Diagnostic performance of diffusion-weighted Mr imaging for the prediction of treatment response. Radiology (2013) 266(2):531–8. doi: 10.1148/radiol.12120167 23151830

[31] Chen Y Liu X Zheng D Xu L Hong L Xu Y . Diffusion-weighted magnetic resonance imaging for early response assessment of chemoradiotherapy in patients with nasopharyngeal carcinoma. Magn Reson Imaging (2014) 32(6):630–7. doi: 10.1016/j.mri.2014.02.009 24703576

[32] Zheng X Chen Y Zheng D Xiao Y Chen J Pan J . Diffusion kurtosis imaging and tumour microstructure for monitoring response to radiotherapy in human nasopharyngeal carcinoma xenografts. Jpn J Clin Oncol (2020) 50(5):548–55. doi: 10.1093/jjco/hyaa002 32009176

[33] Li YT Cercueil JP Yuan J Chen W Loffroy R Wang YX . Liver intravoxel incoherent motion (Ivim) magnetic resonance imaging: A comprehensive review of published data on normal values and applications for fibrosis and tumor evaluation. Quant Imaging Med Surg (2017) 7(1):59–78. doi: 10.21037/qims.2017.02.03 28275560 PMC5337188

[34] Koopman T Martens RM Lavini C Yaqub M Castelijns JA Boellaard R . Repeatability of arterial input functions and kinetic parameters in muscle obtained by dynamic contrast enhanced Mr imaging of the head and neck. Magn Reson Imaging (2020) 68:1–8. doi: 10.1016/j.mri.2020.01.010 31978517

[35] Zheng D Yue Q Ren W Liu M Zhang X Lin H . Early responses assessment of neoadjuvant chemotherapy in nasopharyngeal carcinoma by serial dynamic contrast-enhanced Mr imaging. Magn Reson Imaging (2017) 35:125–31. doi: 10.1016/j.mri.2016.08.011 27587228

[36] Qin Y Yu X Hou J Hu Y Li F Wen L . Prognostic value of the pretreatment primary lesion quantitative dynamic contrast-enhanced magnetic resonance imaging for nasopharyngeal carcinoma. Acad Radiol (2019) 26(11):1473–82. doi: 10.1016/j.acra.2019.01.021 30772137

[37] Zhang H Li W Fu C Grimm R Chen Z Zhang W . Comparison of intravoxel incoherent motion imaging, diffusion kurtosis imaging, and conventional dwi in predicting the chemotherapeutic response of colorectal liver metastases. Eur J Radiol (2020) 130:109149. doi: 10.1016/j.ejrad.2020.109149 32659615

[38] Wong KH Panek R Dunlop A McQuaid D Riddell A Welsh LC . Changes in multimodality functional imaging parameters early during chemoradiation predict treatment response in patients with locally advanced head and neck cancer. Eur J Nucl Med Mol Imaging (2018) 45(5):759–67. doi: 10.1007/s00259-017-3890-2 PMC5978912 29164301

