# Supplementary material for: A comparative study of functional MRI in predicting response of regional nodes to induction chemotherapy in patients with nasopharyngeal carcinoma
Source: Front Oncol. 2022 Aug 31;12:960490. doi: 10.3389/fonc.2022.960490 (PMC9472652; doi:10.3389/fonc.2022.960490)
Supplement: Supplementary file 1 [file Table_1.docx]

Supplementary table 1. MRI standard protocols.

|  | **T2-weighted** | **DWI** | **DKI** | **IVIM** | **DCE** |
| --- | --- | --- | --- | --- | --- |
| **TR** | 5299.0 | 2000.0 | 3500.0 | 4000 | 5.0 |
| **TE** | 68.0 | 67.9 | 86.8 | 73.3 | 1.2 |
| **Slice** **thickness** | 6.0 | 6.0 | 6.0 | 6.0 | 5.0 |
| **Slice gap** | 1.0 | 1.0 | 1.0 | 1.0 | 1.0 |
| **Fov(**mm^2^**)** | 180×240 | 180×240 | 220×220 | 220×220 | 300×300 |
| **Bandwidth** | 62.50 | 250.0 | 250.0 | 250.0 | 125.0 |
| **b values**  **(**mm^2^/s**)** |  | (0, 600) | (0,500, 1000, 2000) | (0,25,50,75,100,150  ,200,500,800, 1000) |  |
| **Pixel size** | 0.7×0.9 | 1.7×1.7 | 2.3×1.7 | 2.3×1.7 | 1.6×1.9 |
| **Echo train length** |  | 128 | 128 | 128 |  |
| **Time (min)** | 4:30 | 2:00 | 4:09 | 2:44 | 4:44 |

Abbreviations: DKI: diffusion kurtosis imaging; IVIM: intravoxel incoherent motion; DCE-MRI:

dynamic contrast enhanced magnetic resonance imaging.
